# Tenosinovitis infecciosa como foco inusual de shock séptico: presentación de un caso

**DOI:** 10.1016/j.aprim.2025.103393

**Published:** 2025-11-06

**Authors:** María Mora-Aznar, Rubén Ferrer-Sorolla, Valentín del Villar-Sordo

**Affiliations:** a Unidad de Cuidados Intensivos, Hospital Nuestra Señora de Gracia, Zaragoza, España; b Centro de Salud de Aliaga, Teruel, España; c Departamento de Medicina, Dermatología y Toxicología, Facultad de Ciencias de la Salud, Universidad de Valladolid, Soria, España

## Introducción

El shock séptico representa una de las complicaciones más graves de la sepsis y constituye una causa importante de morbimortalidad en pacientes hospitalizados, especialmente en población anciana. El origen más habitual de la sepsis suele localizarse en los aparatos respiratorio, urinario o digestivo; sin embargo, los focos osteoarticulares e infecciosos de partes blandas son menos frecuentes y pueden pasar desapercibidos.

La tenosinovitis séptica es una infección infrecuente que afecta a la vaina sinovial de los tendones y que, de no tratarse precozmente, puede progresar a complicaciones graves como la destrucción articular, la extensión a tejidos vecinos o incluso la bacteriemia, con desarrollo de sepsis y shock séptico^1^, ^2^. Entre los microorganismos causales, *Staphylococcus aureus* ocupa un lugar predominante, y su identificación precoz es fundamental para instaurar una antibioterapia dirigida y medidas quirúrgicas de control del foco si es preciso^3^, ^4^.

## Material y Métodos

Análisis retrospectivo de caso entre conjunto de enfermos incluidos en el proyecto “Evaluación y seguimiento tras ingreso prolongado en UCI, y perspectivas de control. Área de Salud de Soria”, entre 2012-2020, en las Unidades de Cuidados Intensivos (UCI) del Hospital Santa Bárbara de Soria y del Hospital Nuestra Señora de Gracia de Zaragoza.

## Resultados

**Figura 1:**
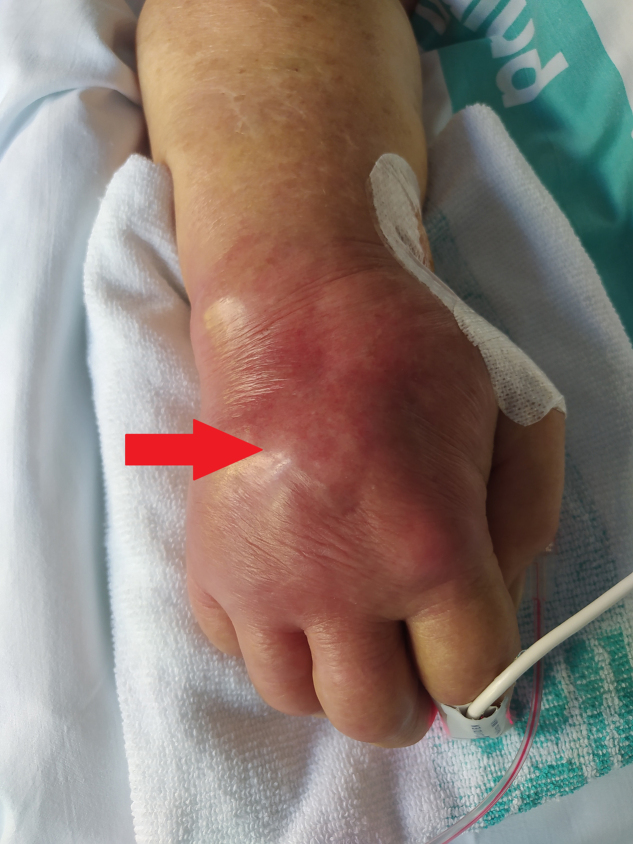
Tenosinovitis infecciosa. Visión directa del dorso de la mano derecha de la paciente, enrojecida y tumefacta como signo indirecto de tenosinovitis subyacente.

Se presenta el caso de una mujer de 82 años, inmunocompetente, con antecedentes médicos de hipertensión arterial, dislipidemia y artrosis generalizada, en tratamiento domiciliario con enalapril/hidroclorotiazida 20 mg/12,5 mg un comprimido cada 24 h, buprenorfina 35 μg/h un parche cada 72 h, simvastatina 20 mg un comprimido cada 24 h y pregabalina 75 mg un comprimido cada 12 h. Valorada en sucesivas ocasiones en atención primaria por dolor y tumefacción de muñeca derecha (resistente a tratamiento con corticoides y analgésicos intramusculares y orales, respectivamente) asociado a uso recurrente de bastón, con diagnóstico inicial de tenosinovitis (fig. 1); en los últimos días se han sumado fiebre, dolores generalizados, disminución del nivel de conciencia y debilidad con postración. Es derivada al servicio de urgencias del hospital de referencia, donde se objetivan signos y síntomas compatibles con shock séptico sin foco, descartando como origen del mismo causa respiratoria, digestiva y urinaria tras nueva anamnesis y realización de TAC torácica, ecografía abdominal y urocultivo negativos. Al tratarse de una situación crítica con inestabilidad hemodinámica y una puntuación en la escala de Glasgow de 6, se inicia tratamiento con fluidoterapia intensiva sin respuesta, con necesidad de administración de noradrenalina en perfusión continua hasta 0,08 μg/kg/min; se procede a intubación orotraqueal urgente con sedoanalgesia intravenosa profunda.

**Figura 2:**
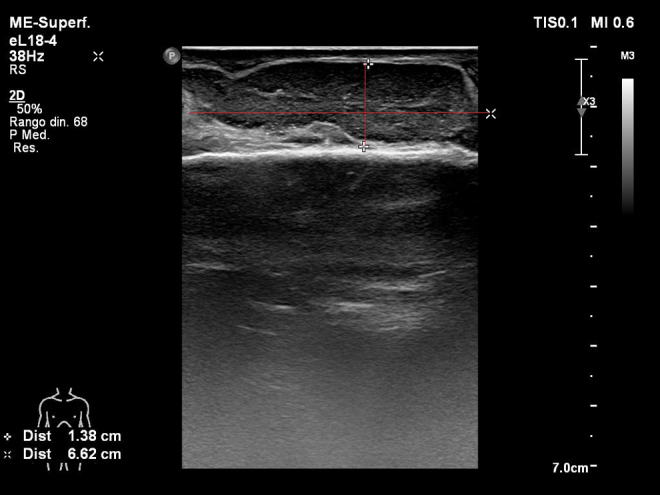
Imagen ecográfica de la superficie dorsal de la mano derecha; se aprecia el tendón extensor común de los dedos, de espesor y ecoestructura normales, rodeado de material hipoecogénico, móvil, en un segmento de unos 6,62 cm de longitud y 1,38 cm de espesor, con hipervascularización de la sinovial.

Trasladada a la unidad de cuidados intensivos, se inicia antibioterapia intravenosa de amplio espectro (meropenem + amikacina + linezolid) previa toma de hemocultivos; al objetivar como posible foco infeccioso tenosinovitis séptica en la mano derecha, se modifica por meropenem con daptomicina intravenosa y se solicita interconsulta a traumatología para valoración y realización de ecografía de partes blandas (fig. 2). Se realiza drenaje con limpieza por traumatología en la propia unidad de cuidados intensivos, con incisión en el dorso de la mano, drenaje de abundante débito hemopurulento, toma de muestras para estudio microbiológico, limpieza con agua oxigenada y povidona yodada, y mantenimiento de drenaje tipo Penrose con curas diarias durante 5 días. Se objetiva aislamiento en hemocultivos y muestras de la herida de *S. aureus* sensible a meticilina (SASM), por lo que se desescala antibiótico a cloxacilina intravenosa. Presenta una mejoría progresiva de los síntomas y signos analíticos de shock séptico. Se consigue la estabilización de la paciente, por lo que se logra despertar, y se procede a la extubación al quinto día de ingreso en la unidad de cuidados intensivos. Los controles microbiológicos posteriores fueron negativos. Se realizó un ecocardiograma de control para descartar endocarditis infecciosa secundaria a bacteriemia por SASM. Se dio de alta a la planta de hospitalización convencional de traumatología con el diagnóstico de shock séptico secundario a tenosinovitis infecciosa en mano derecha por SASM.

## Discusión

El manejo exitoso del caso precisó precisó de una perspectiva longitudinal del mismo, más allá de los límites físicos de la UCI, y del abordaje transversal, con la colaboración entre medicina de familia, cuidados intensivos, traumatología y microbiología, destacando el valor de la coordinación clínica para estabilizar a la paciente y controlar el foco infeccioso. La identificación del agente causal (SASM), junto con el drenaje quirúrgico y la desescalada antibiótica, fueron determinantes para la recuperación favorable de la paciente.

## Financiación

Se declara que no se ha recibido financiación alguna ni privada ni pública para la realización de este estudio.

## Consideraciones éticas

El escrito fue aprobado por el Comité de Ética de Investigación de Soria, con n.^o^ de referencia: 42-0249. Se obtuvo el consentimiento informado de la paciente.

## Conflicto de intereses

Los autores declaran no tener ningún conflicto de intereses.
